# Supplementary material for: Design of Two New Sulfur Derivatives of Perezone: In Silico Study Simulation Targeting PARP-1 and In Vitro Study Validation Using Cancer Cell Lines
Source: Int J Mol Sci. 2024 Jan 10;25(2):868. doi: 10.3390/ijms25020868 (PMC10815500; doi:10.3390/ijms25020868)
Supplement: Supplementary file 1 [file ijms-25-00868-s001.zip › ijms-2800374-supplementary.pdf]

Design of two new sulfur derivatives of perezone: In silico study simulation targeting PARP-1, and in vitro study validation using cancer cell lines.

Alejandro Rubiales-Martínez, Joel Martínez, Elvia Mera-Jiménez, Javier Pérez-Flores, Guillermo Téllez-Isaías, René Miranda Ruvalcaba, Maricarmen Hernández-Rodríguez, Teresa Mancilla Percino, Martha Edith Macías Pérez, and María Inés Nicolás-Vázquez

Content

Table S1. Amino acid residues that provide the recognition of studied compounds **1-4** by PARP-1 obtained by analysis at different times of MD simulations.[1]

Table S2. Atomic charges of some atoms for **1**, **2**, **3**, and **4** molecules, in e^-.

Table S3. Recognition among amino acid residues of PARP-1 and **8-10** molecules.[1]

Figure S1. Cytotoxic activity of compounds **8** (white), **9** (black), and **10** (gray), in the cancer cell lines A549, MDA-MB-231, Hela, and MCF-7, employing perezone (dotted) as a control. Cell from each line were incubated without control and with increased concentrations of the studied compounds during 48 h (n= 8). At the end of the incubation, an MTT assay was performed to determine the viability of cells under treatment. Each bar in the chart indicates the percentage means and standard error of the mean.

Figure S2: Cytotoxic activity of compounds **8** (white), **9** (black), and **10** (gray), in healthy endothelial cells, VERO cells, and mixed culture of microglia, employing perezone (dotted) as a control. Cells from each line were incubated without control and with increased concentrations of the studied compounds during 48 h (n= 8). At the end of the incubation, an MTT assay was performed to determine the viability of cells under treatment. Each bar in the chart indicates the percentage means and standard error of the mean.

Table S1. Amino acid residues that provide the recognition of studied compounds **1-4** by PARP-1 obtained by analysis at different times of MD simulations.[1]

Time (ns)	Compounds			
	**1**	**2**	**3**	**4**
10	Tyr246, Asn106, Gly202, Tyr228, Gly227, Gln098, Val101, Gly202, **Tyr235**	Asp105, Ile211, Gly227, Tyr228, **Hys201**, Ser203, Tyr246, Lys242, Ala219, Phe236, **Tyr235**, Ile234	Asn106, Tyr246, Glu102, Ala237, Trp200, Leu199, **Tyr235**, **Hys201**, Trp200, Gly202, Ser203	Gly202, Asn106, Ala219, Ile211, Leu216, Ile234, **Hys201**, Tyr246, Hys248, Ile333, **Tyr235**
30	Thr163, Ala162, Hys161, Ser241, **Tyr235**, Leu324, Thr164	Tyr228, Asn207, Arg217, Ser203, Gly215, Tyr246, **Hys201**, Gly202, Tyr331, **Tyr235**	Asp106, Asp105, Asn207, Ile211, Ser203, Gly202, Val101, Gly227, **Tyr235**, Tyr246	Gly233, Gln098, Val101, Asp105, Ala223, Pro220, Tyr246, Gly102, Tyr331, **Tyr235**
50	Ser250, Thr249, Pro254, Phe348, Asn347, Tyr168, Ala167, Asn347	Tyr246, Ser202 Gly233, Asn106, Gly210, Ile211, Asp105, Tyr228, Ser203, Gly202, **Hys201**, **Tyr235**, Asn207, Ile234, Arg217	Asp105, **Hys201**, Glu102, **Tyr235**, Val101, Ser203, Gly202, Tyr228, Gly227	Val101, Tyr049, Ala223, Ala219, Ile218, Arg217, Tyr331, **Hys201**, **Tyr235**, Tyr246, Tyr228, Lys232, Ile234, Tyr246, Ser203, Gly233

[1] The highlighted amino acid residues correspond to the catalytic triad.

Table S2. Atomic charges of some atoms for **1**, **2**, **3**, and **4** molecules, in e^-.

Atoms	Compounds			
	**1**	**2**	**3**	**4**
**C_1**	0.482	0.495	0.480	0.486
**C_2**	-0.042	-0.064	-0.160	-0.120
**C_3**	0.338	0.277	0.342	0.299
**C_4**	0.465	0.484	0.475	0.485
**C_5**	-0.025	-0.026	-0.165	-0.100
**C_6**	-0.173	-0.219	-0.320	-0.319
**C_7**	-0.609	-0.602	-0.598	-0.500
**C_8**	-0.247	-0.245	-0.237	-0.247

**O$_1$**	-0.494	-0.521	-0.578	-0.522
**O$_2$**	-0.507	-0.507	-0.573	-0.559
**O$_3$**	-0.595	-0.559	-0.642	-0.654
**O$_4$**	-	-	-0.641	-0.561
**H$_1$**	0.508	-	0.497	0.493
**H$_2$**	0.236	0.218	-	-

Table S3. Recognition among amino acid residues of PARP-1 and **8-10** molecules.[1]

Compounds	Amino acid residue
**8**	**Tyr235**, Arg217, Trp200, **Hys201**, Phe236, Ser203, Trp200, Ala237, Gly202, Tyr246
**9**	Tyr228, **Hys201**, Arg217, **Tyr235**, Tyr228, Phe236, Ser243, Tyr246, Ile234, Ala237
**10**	Gly202, Gly233, Lys242, **Hys201**, **Tyr235**, Ile234, Ser203, Tyr246

[1] The highlighted amino acid residues correspond to the catalytic triad.

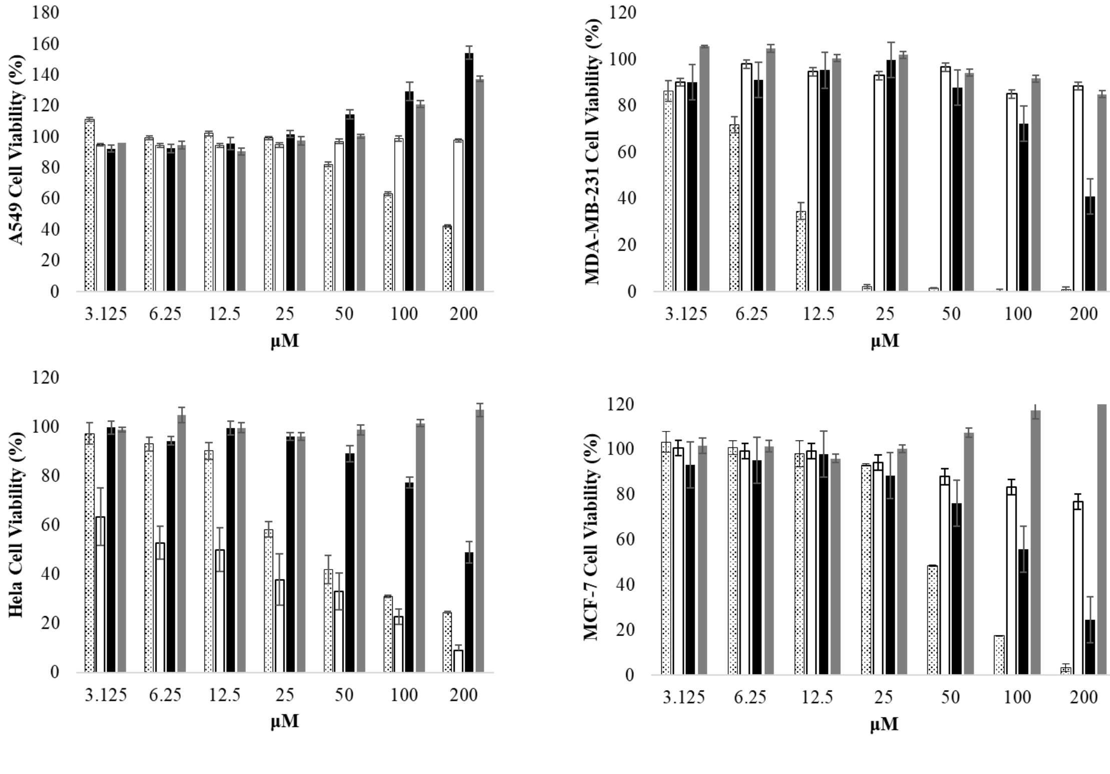

Figure S1. Cytotoxic activity of compounds **8** (white), **9** (black), and **10** (gray), in the cancer cell lines A549, MDA-MB-231, Hela, and MCF-7, employing perezone (dotted) as a control. Cell from each line were incubated without control and with increased concentrations of the studied compounds during 48 h (n= 8). At the end

of the incubation, an MTT assay was performed to determine the viability of cells under treatment. Each bar in the chart indicates the percentage means and standard error of the mean.

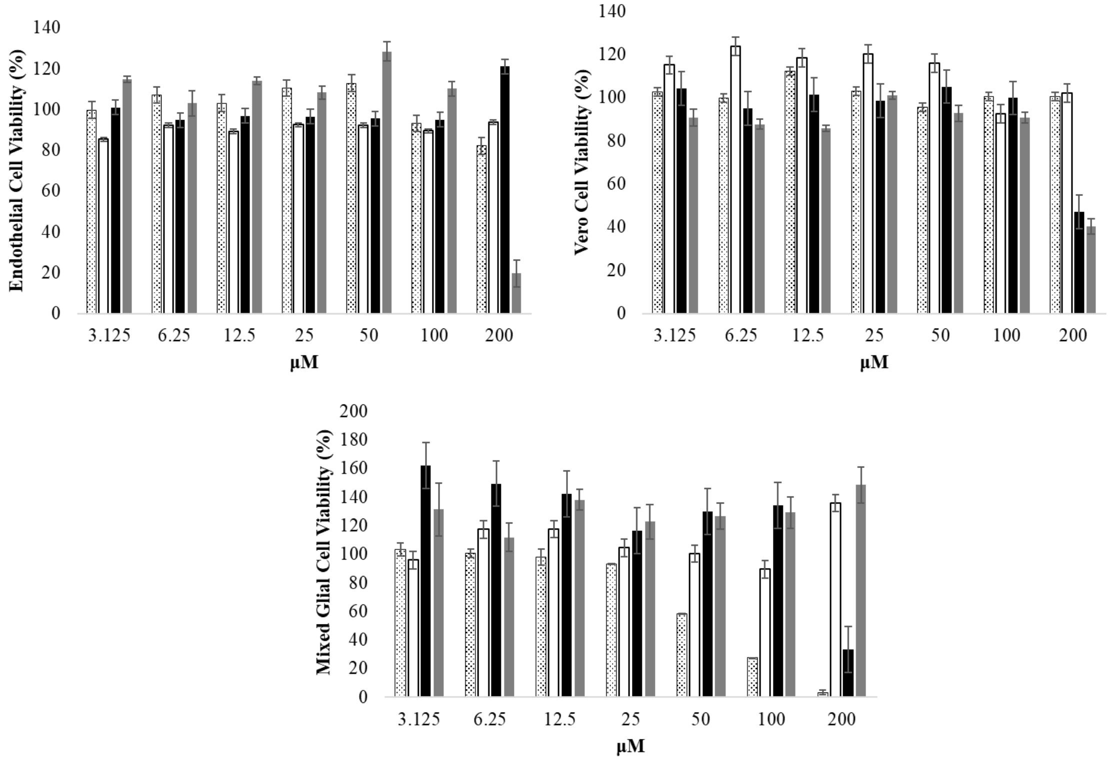

Figure S2: Cytotoxic activity of compounds **8** (white), **9** (black), and **10** (gray), in healthy endothelial cells, VERO cells, and mixed culture of microglia, employing perezone (dotted) as a control. Cells from each line were incubated without control and with increased concentrations of the studied compounds during 48 h (n= 8). At the end of the incubation, an MTT assay was performed to determine the viability of cells under treatment. Each bar in the chart indicates the percentage means and standard error of the mean.
